# Autoimmune Glial Fibrillary Acidic Protein Astrocytopathy: A Review of the Literature

**DOI:** 10.3389/fimmu.2018.02802

**Published:** 2018-12-05

**Authors:** Fulan Shan, Youming Long, Wei Qiu

**Affiliations:** ^1^Department of Neurology, The Second Affiliated Hospital of Guangzhou Medical University, Guangzhou, China; ^2^Key Laboratory of Neurogenetics and Channelopathies of Guangdong Province and the Ministry of Education of China, Collaborative Innovation Center for Neurogenetics and Channelopathies, Institute of Neuroscience and The Second Affiliated Hospital of Guangzhou Medical University, Guangzhou, China; ^3^Department of Neurology, Zengcheng District People's Hospital of Guangzhou, Guangzhou, China; ^4^Department of Neurology, The Third Affiliated Hospital of Sun Yat-Sen University, Guangzhou, China

**Keywords:** astrocyte, antibody, meningoencephalitis, glial fibrillary acidic protein, astrocytopathy

## Abstract

Autoimmune glial fibrillary acidic protein (GFAP) astrocytopathy is an autoimmune disease of the nervous system first defined in 2016. GFAP autoantibody, especially IgG that binds to GFAPα, has been reported in the cerebrospinal fluid (CSF) and serum of patients with GFAP astrocytopathy. The positive predictive value of GFAP antibody in the CSF is higher than in the serum. Tissue-based assay (TBA) and cell-based assay (CBA) are both recommended methods for the detection of GFAP antibody. GFAP astrocytopathy is accompanied by neoplasms, but the relationship between virus infection and GFAP astrocytopathy is unclear. GFAP antibody itself does not induce pathological changes; it is only a biomarker for the process of immune inflammation. The pathology of GFAP astrocytopathy in humans is heterogeneous. GFAP astrocytopathy is commonly diagnosed in individuals over 40 years old and most patients have an acute or subacute onset. Clinical manifestations include fever, headache, encephalopathy, involuntary movement, myelitis, and abnormal vision. Lesions involve the subcortical white matter, basal ganglia, hypothalamus, brainstem, cerebellum, and spinal cord. The characteristic MRI feature is brain linear perivascular radial gadolinium enhancement in the white matter perpendicular to the ventricle. Currently, there are no uniform diagnostic criteria or consensus for GFAP astrocytopathy and coexisting neural autoantibodies detected in the same patient make the diagnosis difficult. A standard treatment regimen is yet to be developed. Most GFAP astrocytopathy patients respond well to steroid therapy although some patients are prone to relapse or even die.

## Background

The novel concept of astrocytopathy, including neuromyelitis optica spectrum disorders (NMOSD) and autoimmune glial fibrillary acidic protein (GFAP) astrocytopathy, was recently suggested (1, 2). Unlike NMOSD characterized by aquaporin (AQP) 4 antibody, GFAP astrocytopathy is a meningoencephalomyelitis or limited form of meningoencephalomyelitis associated with IgG binding to GFAP. This disease usually involves the cerebra, meninges, spinal cord and optic nerve, and manifests as fever, headache, encephalopathy, myelitis, and abnormal vision (2–13).

## History

Since 1991, reports of the clinical manifestations, images, and features of cerebrospinal fluid (CSF) in corticosteroid-responsive meningoencephalomyelitis, also known as chronic or subacute corticosteroid-responsive non-vasculitic autoimmune inflammatory meningoencephalitis (NAIM), have been published (14). Patients suffer from NAIM manifested as chronic/subacute encephalopathy or progressive dementia, and they tend to have severe abnormal findings by electroencephalography but no obvious changes by magnetic resonance imaging (MRI). Pathological analysis has revealed periangitis, gliosis, and T and B cell infiltration, with intact blood vessels in the brain parenchyma. As an autoimmune disease, NAIM is very sensitive to corticosteroid treatment.

Reports of zoonotic autoimmune disease are increasing. For example, N-methyl-D-aspartic acid (NMDA) antibody encephalitis was reported in polar bears (15). In addition, GFAP antibody was confirmed as a biomarker for necrotizing meningoencephalitis of pug dogs (16, 17). Classification by pathology includes granulomatous meningoencephalomyelitis (GME), necrotizing meningoencephalitis (NME), and necrotizing leukoencephalitis (NLE).

In 2016, a group led by Lennon (2, 3) in the Mayo Clinic published two important reports of meningoencephalitis in humans and termed the disorder autoimmune GFAP astrocytopathy. Our group started similar studies in 2013 and reported the pathological features of several cases of GFAP astrocytopathy. A long follow-up study has also been carried out. Several studies of GFAP astrocytopathy have been published to date (2–13) (Table 1).

**Table 1:** Literatures of human GFAP astocytopathy.

**Author**	**Year**	**Country**	***n***	**Pathological examination**	**Main findings**
Fang et al. (2)	2016	USA	16	No	The first paper to described human GFAP astrocytopathy. They describe GFAP-IgG found in serum or cerebrospinal fluid that is specific for a cytosolic intermediate filament protein of astrocytes.
Flanagan et al. (3)	2017	USA	102	Yes	Retrospectively analyzed 102 GFAP-IgG positive patients. Specificity of serum and CSF testing, the clinical and radiological phenotype evaluate, the significance of coexisting antibodies, and therapy responses were reported. CSF-GFAPαa-IgG is highly specific for an immunotherapy-responsive autoimmune CNS disorder.
Yang et al. (4)	2017	China	7	Yes	To assess the treatment response in seven GFAP-IgG-positive patients with long-term follow-up. Some patients with GFAP astrocytopathy experienced a poor response to treatment although they received steroids and immunosuppressive agents,
Long et al. (5)	2018	China	19	Yes	To describe the clinical, radiological and pathological features in 19 patients with CSF-GFAP-IgG in CSF. The features of the neuropathology and immunopathology of GFAP astrocytopathies were perivascular inflammation and loss of astrocytes and neurons.
Yang et al. (6)	2018	China	10	Yes	To study overlapping syndromes in autoimmune GFAP astrocytopathy. Overlapping antibodies are common in GFAP astrocytopathy.
Iorio et al. (7)	2018	Italy	22	Yes	To report the clinical and immunological characteristics of 22 new patients with GFAP-IgG. GFAP autoimmunity is not rare. The clinical spectrum encompasses meningoencephalitis, myelitis, movement disorders, epilepsy and cerebellar ataxia. coexisting neurological and systemic autoimmunity are relatively common. Immunotherapy is beneficial in most cases.
Zarkali et al. (8)	2018	UK	1	No	To report a young man presenting with subacute meningoencephalitis and subsequent myelitis, and discuss the typical presentation and management of this severe but treatable condition.
Shu et al. (9)	2018	China	1	Yes	To examined brain biopsy sections from a patient with autoimmune GFAP astrocytopathy using hematoxylin and eosin and Luxol fast blue staining, and immunostaining with antibodies.
Martin et al. (10)	2018	USA	1	Yes	To report a 13-years-old girl with acute-onset meningoencephalitis and incidental finding of ovarian teratoma was found to have coexisting anti-NMDA-R and GFAP antibodies present in her cerebrospinal fluid.
Li et al. (11)	2018	China	1	No	To report a case of autoimmune GFAP astrocytopathy after herpes simplex viral encephalitis.
Dubey et al. (13)Sechi et al. (18)	20182018	USAUSA	90 13	YesNo	This study demonstrates CSF GFAPα-IgG is a specific autoimmune meningoencephalomyelitis biomarker, with favorable corticosteroid response. Lack of response should prompt evaluation for co-existing NMDA-R-IgG or malignancy. This study found that spinal cord lesions in GFAP-IgG myelitis were commonly longitudinally extensive (≥80%) and centrally located. Compare to AQP4-IgG lesions, they were more subtle lesions with poorly defined margins and less swelling. In GFAP-IgG myelitis, spinal cord central canal, punctate or leptomeningeal enhancement was typical.

## Detection Method

AQP4 expressed on the endfeet of astrocytes was identified as a biomarker of NMOSD. The methods to detect this antibody include tissue-based assay (TBA), cell-based assay (CBA), flow cytometry, immunoblotting, and immunoprecipitation assay. Because GFAP is a cytosolic intermediate filament protein of astrocytes, methods for the detection of its antibody are limited. Currently, we can test for GFAP antibody by IF, CBA, and western blot.

**Figure 1:**
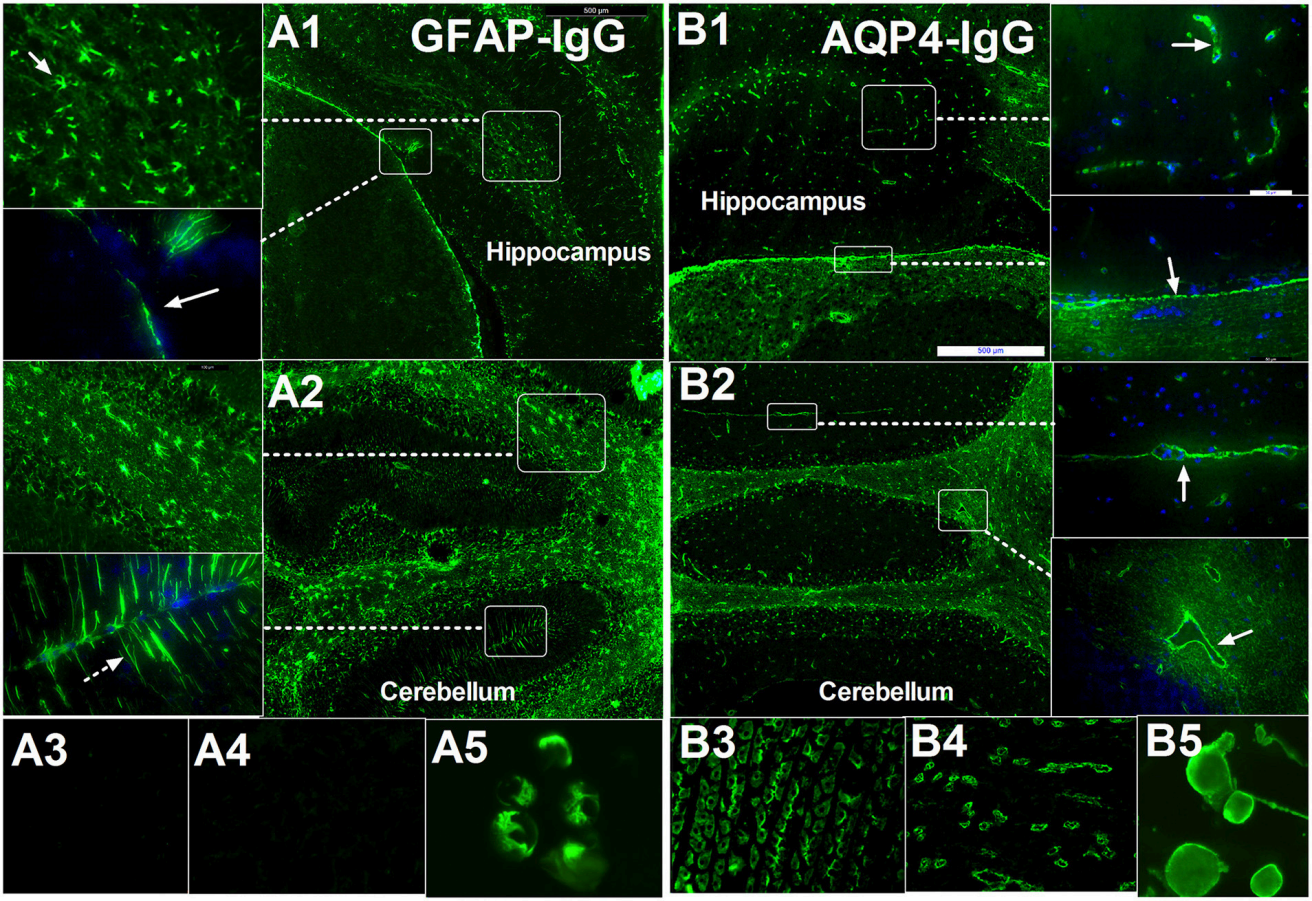
Comparison of immunofluorescence-pattern between GFAP-IgG and AQP4-IgG. **(A1–A5)** IgG from patient with GFAP astrocytopathy. **(A1)** The IgG is bound to the foot process at pia (arrow) and astrocyte body in hippocampus. **(A2)** IF pattern in different layer of cerebellum: (1) The IgG is bound to the astrocyte body in different layer, especially white matter (arrow); (2) it was detected in molecular layers with bergmann radial pattern (arrow). **(A3,A4)** no pattern-specific staining was detected in the kidney and stomach tissue but positive for GFAP-transinfected HEK-293 cell. **(B1–B5)** IgG from a positive AQP4-IgG NMO patient. **(B1)** The IgG is bound to the cell with the foot process around the microvessels (arrow) in brain and pia (arrow). **(B2)** Anti-AQP4 pattern located at the border between two molecular layers with Virchow-Robin space profile (arrow) without bergmann radial pattern. **(B3–B5)** Pattern-specific staining was detected in the stomach, kidney tissue, and AQP4-transinfected HEK-293 cell.

Our previous studies (4, 5) and unpublished data used frozen sections of the hippocampus, brainstem and cerebellum and demonstrated that a characteristic IF pattern of GFAP-IgG was observed when IgG binds to the pia and subpia mimicking AQP4-IgG (Figure 1). However, the IF pattern differs from AQP4-IgG as follows (Figure 1): (1) it is prominent in the cell body and end-process located in all layers (molecular layer, white matter, and granular layer) whereas AQP4-IgG mainly binds to locations around the microvessels and the Virchow-Robin space; (2) in the cerebellum, it was detected in the molecular layers with a Bergmann radial pattern (Figure 1A2), which differs from the AQP4-IgG pattern that only has a microvessel profile (Figures 1B1,B2). Furthermore, the AQP4-IgG pattern is located at the border between two molecular layers with a Virchow-Robin space profile (Figure 1B2); and (3) in contrast to AQP4-IgG, no pattern-specific staining was detected in the stomach or kidney tissues when using CSF (Figure 1).

Tissue sections from different animals affect the results. In our study, sections from rats are better than from monkeys. Methods based on histology are sensitive but they cannot distinguish different subtypes of GFAP antibody. A disadvantage of this method is that it detects other protein antibodies coexpressed with GFAP in astrocytes, resulting in false positive results. For example, transglutaminase-6 is expressed in astrocytes, and its antibody is related to progressive multiple sclerosis (19).

CBA (Figure 1B5) is recommended for testing for AQP4 antibody and is the main method to test GFAP antibody. Because GFAP protein has more than eight isomers, using CBA to test all these isomer antibodies is very difficult. Testing of GFAP-IgG in the Mayo Clinic reported IgG reactive with the GFAPα isoform in 102 patients (100%) and GFAP-reactive IgG was lower than for other isoforms (3). A series of 22 patients from a European tertiary referral hospital reported a frequency of 100% binding to GFAPα, 63.6% to GFAPα and GFAPδ, and none to the GFAPδ isoform alone (7). Therefore, detection of GFAPα may be performed by CBA. However, in 19 Chinese patients who underwent CSF testing, we found fourteen cases that were GFAPα-IgG positive, and five cases who were only GFAPε-IgG positive, indicating that this result should be confirmed by other laboratories in the future.

Regarding the specificity of GFAP antibodies in the serum, more data are required for evaluation. Early studies using Enzyme-linked Immunosorbent Assay (ELISA) showed high concentrations of GFAP antibody in the serum of patients with Alzheimer's disease and trauma (20, 21). Therefore, the consequence of GFAP antibody in the serum of GFAP astrocytopathy is hard to determine. From our study and other recent reports, GFAP antibody in the CSF had a high specificity and sensitivity (2, 3, 5). Because ELISA has a number of disadvantages, such as low specificity for the detection of antibodies in some neuroimmune diseases (22), the influence of the methodology on the results cannot be ignored.

Earlier literature showed that GFAP antibodies were present in the CSF of some patients with MS (23). It was speculated retrospectively that patients previously diagnosed as MS but who did not meet the latest diagnosis criteria might have GFAP astrocytopathy. According to published studies (Table 1), patients with meningoencephalitis, myelomeningoencephalitis, encephalitis, myelitis, optic neuritis or autonomic nervous dysfunction with unclear reason should be tested for GFAP-IgG. TBA and CBA are both recommended methods.

## Epidemiology and Demographics

GFAP astrocytopathy is a rare condition even if probably under-diagnoses yet. Nowadays, epidemiological data of this disease is limited. GFAP astrocytopathy has a slight female predominance and tends to present in patients over 40 years old. Recently, children with GFAP astrocytopathy were reported with pediatric clinical presentations similar to adults (13). In a Chinese population, 19 patients were positive for GFAP antibodies, comprising 13 females and six males (ratio 2.17). The median age at disease onset was 54 years (range 23–73 years). A study by the Mayo Clinic reported 54% (55/102) were female and the median age at neurologic symptom onset was 44 years (range, 8–103 years). Iorio et al. found that the median age of their patients was 52 years (range: 6–80 years) and 13 patients were female (59%).

## Etiology and Pathogenesis

Currently, there is limited information regarding GFAP astrocytopathy and its pathogenesis. Similar to autoimmune encephalitis, GFAP astrocytopathy is also accompanied by a neoplasm. In a study by Lennon (2, 3), neoplasia was diagnosed in 34% patients, 66% of which were found within 2 years after neurologic onset. Twenty-two patients had neoplasms at disease onset, of which 15 were teratoma of the ovary, three were adrenal carcinoma, two were glioma, one was squamous cell carcinoma, and one was multiple myeloma. Results from Iorio et al. (7) showed that three patients had a history of tumors, and in our recent study, we found one patient with thyroid carcinoma, two patients with suspected meningeoma, and three with other benign tumors. Immunohistochemical staining of an ovarian teratoma from a GFAP astrocytopathy patient showed the cytoplasm of the glial process in neuronal tissue and epithelial cells reacted strongly with GFAP-IgG (7, 13). This supports the concept that ectopic expression in nervous system tissues contributes to the triggering of immune responses, such as that in autoimmune encephalitis (24).

Regarding infection, Lennon (2, 3) reported that 29% patients with GFAP antibody developed flu before neurologic symptoms and one patient had HIV. In a study by Iorio et al. (7), six (27%) patients had a premonitory symptom of flu, including one case of dengue fever. Two researchers from China found evidence of herpes simplex infection in some patients (5, 11). The relationship between virus infection and GFAP astrocytopathy is unclear.

Currently, it is recognized that AQP4 antibody causes the loss of AQP4 antigen expression and the decrease of astrocyte numbers. AQP4 antibody binds to AQP4 expressed in the endfeet of astrocytes and activates complement. The downstream pathways involve excitatory amino acid transporter-2 endocytosis and result in demyelination and tissue necrosis. Using immunohistochemical analysis, we found the loss of AQP4 antigen expression and the decrease of astrocytes at different degrees in the lesions of patients with GFAP astrocytopathy. However, unlike AQP4, GFAP is an intracellular protein, thus it is difficult for GFAP antibody to interact with it. When a patient is positive for AQP4 antibody, the decrease in astrocyte numbers may be caused by AQP4 autoimmunity. However, this might also be secondary to other autoimmune diseases.

Animal model studies (25) indicated that GFAP astrocytopathy is mediated by GFAP peptide–specific cytotoxic T cells. The hypodermic injection of rat cerebral homogenate could induce vacuole or softening lesions in the cerebral cortex and CD3^+^ T cells and microglial cells infiltrate the lesions. GFAP autoantibody can also be detected in the serum when CD3^+^ T cells attach to GFAP-positive astrocytes. These pathological features of rats were consistent with those of canine NME. The above findings indicate that T cell-mediated immunity plays an important role in GFAP astrocytopathy.

## Neuropathology

**Figure 3:**
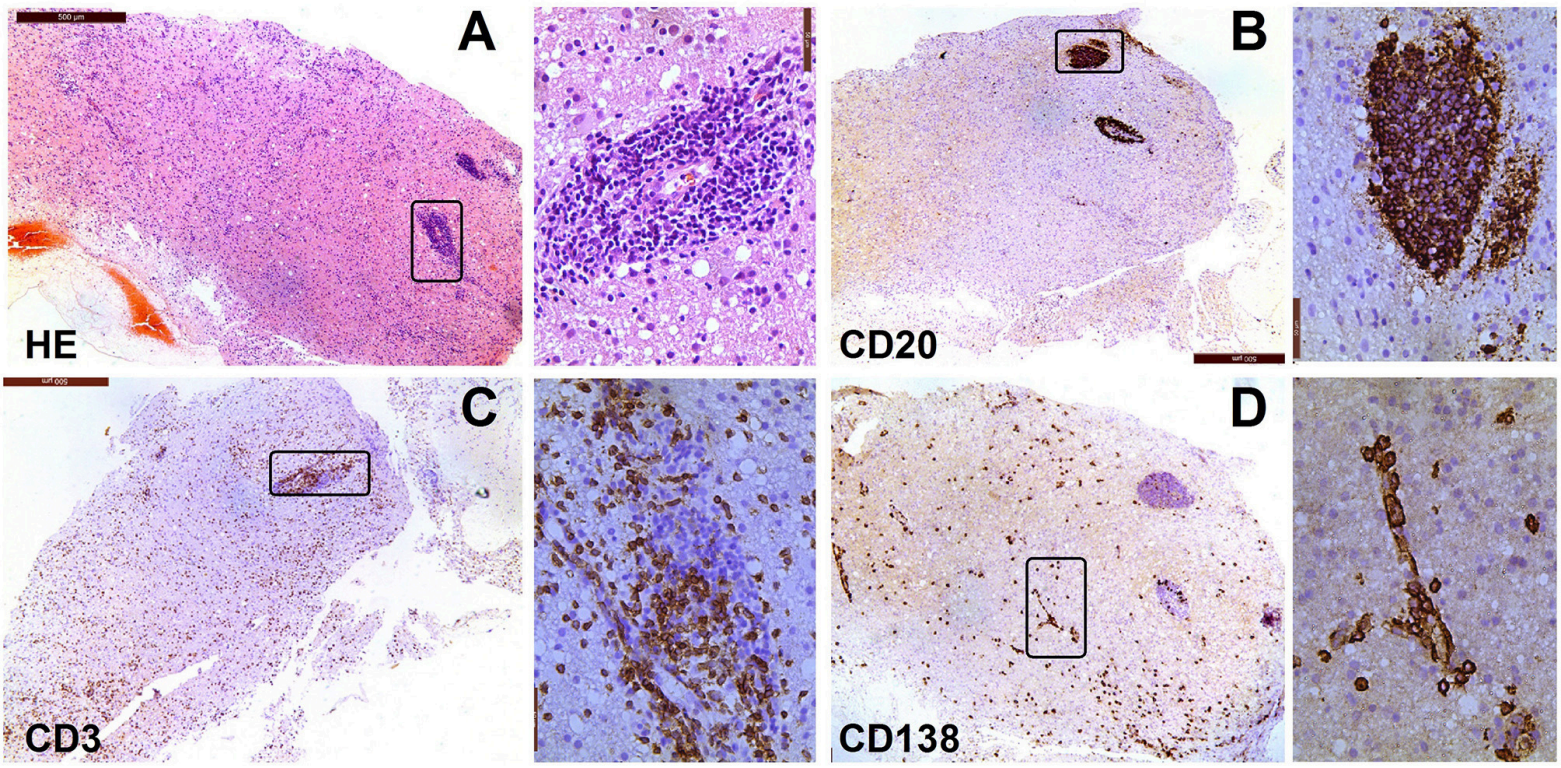
Neuropathological features of a patient with encephalitis. The case involved a woman, whose age at onset was 69 years. In January 2015, she experienced psychosis, dyskinesia, persistent fever, and paralysis of a right-side limb. Antibody detection and biopsy were conducted before the IVMP and IVIG treatment. She then received treatment with methylprednisolone and azathioprine. She did not response well to this treatment and died of acute respiratory failure 1 year later. **(A)** Hematoxylin-eosin staining of brain biopsy tissue shows extensive infiltration of inflammatory cells; **(B)** Immunostaining for anti-CD20 is mainly around the vessels in lesions; **(C)** Immunostaining for anti-CD3 is sparse throughout the lesion, with some staining around the large vessel; **(D)** CD138^+^ cells are present in perivascular, Virchow–Robin, and interstitial spaces.

Histopathological research of human GFAP astrocytopathy is limited. No post-mortem report of this disease has been published. Histopathology analysis of the leptomeningeal biopsy specimen from one patient in a report by Iorio (6) revealed inflammatory changes in local tissues with mononuclear infiltration by macrophages and CD8^+^ T cells. In a study by the Mayo Clinic, there was a rough description of the clinical characteristics including chronic inflammation and microglia activation without vasculitis. In China, five pathological studies reported data from stereotactic biopsy (4, 9). All the studies showed inflammation around small vessels (Figure 3). The vascular wall was unaffected and no necrotic changes were found in tissues. T and B cells had infiltrated and scattered neutrophilic segmented granulocytes and eosinophils were present in the tissue. Many plasma cells were present in two cases. Histochemistry revealed two cases of severe AQP4 and GFAP dislocation, two cases of focal dislocation, and one case without dislocation. Therefore, the pathology of GFAP astrocytopathy in humans is heterogeneous. However, local stereotactic biopsy has disadvantages and does not reflect the whole picture of the disease.

In animal studies, a fatal necrotizing meningoencephalitis with GFAP-IgG as a biomarker was described (25). GME, NME, and NLE are idiopathic inflammatory diseases of the canine central nervous system (CNS) with differences in their clinical and pathological features (13). Histopathological necrosis was observed in both NME and NLE. Regarding the clinical features, GME involved a wide range of tissues including the cerebral cortex, cerebellum, brainstem, optic nerve, and spinal cord, and there was a marked elevation of white blood cells and protein levels in the CSF. Imaging revealed extensive abnormalities in the subcortical white matter. Histopathologically, GME was characterized by the accumulation of CD3^+^ T cells and CD20 B cells around the vessels with an infiltration of plasma cells and monocytes. Neutrophils and granulomas were occasionally observed. Therefore, GME might be an equivalent to autoimmune GFAP astrocytopathy in humans.

## Clinical Features

Most patients have an acute or subacute onset, and progressively aggravated disease or have a relapse-remission pattern. Clinical manifestations include fever, headache, encephalopathy, involuntary movement, myelitis, abnormal vision, ataxia, mental disorder, epilepsy, abnormal autonomic nervous function, and other symptoms and signs of meningoencephalomyelitis. Reports of this disease were presented by the Mayo Clinic (USA), the Second Affiliated Hospital of Guangzhou Medical University (China), and Catholic University (Italy) (2–7, 13).

The report by Lennon (3) included 102 patients, whose most prominent clinical manifestations were encephalitis and meningitis (54.5%), followed by myelitis (10.5%) encephalomyelitis (8%), optic neuropathy, meningitis, ataxia, and meningoencephalomyelitis. Rare symptoms included epilepsy, dementia, and autonomic nervous dysfunction. In the study by Iorio, 10/22 (45%) cases manifested as encephalitis and meningitis. Other symptoms included ataxia, chorea, myelitis, optic neuritis, epilepsy, and dyskinesia (7). However, in a Chinese study, more patients suffered from myelitis (68.4%) and optic neuritis (63.2%), and longitudinally extensive transverse myelitis was more common (5). A recent study showed 10 children with positive GFAP-IgG had similar manifestations to previous studies in adults. Especially, seven cases with positive CSF-IgG all had a meningoencephalitis phenotype (13).

## Biological and Imaging Features

CSF in patients with GFAP astrocytopathy have high numbers of white blood cells (>50 × 106/L), including lymphocytes, monocytes, and multinucleate cells. In addition, protein levels were increased to >1 g/L. The positive predictive value of GFAP antibody in the CSF is higher than in the serum. Patient serum and CSF in three cohorts (2, 3, 5, 7) contained other auto-antibodies, such as NMDAR antibody, AQP4 antibody and other antibodies related to autoimmune diseases.

**Figure 2:**
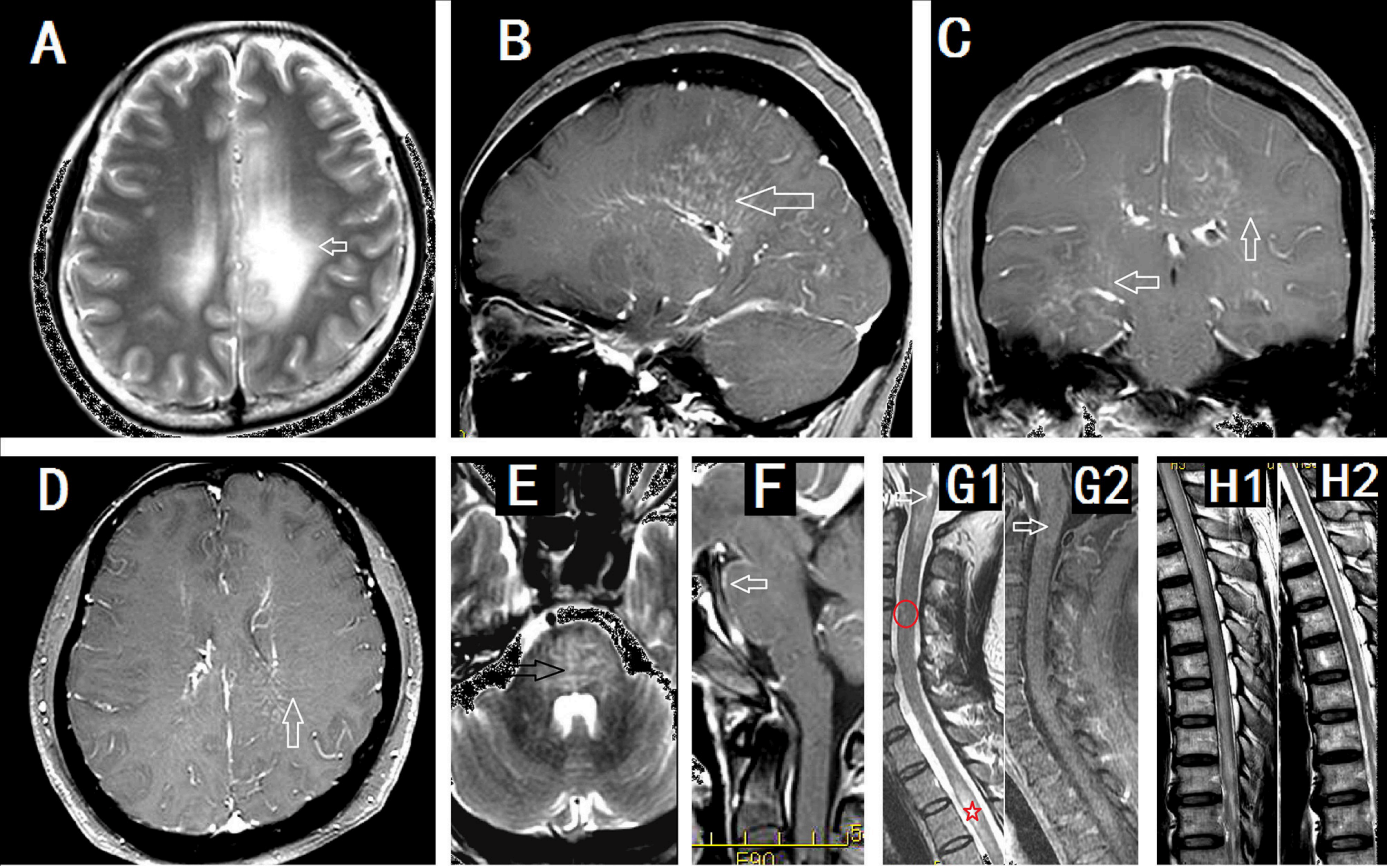
Imaging findings in patients with GFAP astrocytopathy. **(A–D)** were from a female meningoencephalitis patient. **(A)** MR images showing extensive abnormalities in the white matter around the ventricle (arrow). **(B)** Sagittal section showed linear perivascular radial gadolinium enhancement in the white matter perpendicular to the ventricle(arrow). **(C,D)** Coronal section **(C)** and cross section **(D)** showed vessel-like enhancement (arrows). **(E)** and **(F)** from a male meningoencephalitis patient showed pons abnormality (black arrow) and pia enhancement (white arrow). **(G1)** and **(G2)** were from a female with myelitis. **(G1)** Cervical lesion extended to the area postrema of medulla(arrow), sparse cervical abnormality(red round area) and thoracic LESCLs (star marker). **(G2)** Slightly enhancement in medulla (arrow). **(H1)** and **(H2)** were from a male meningoencephalomyelitis patient. Longitudinal extensive lesions in the whole spinal cord **(H1)** and soon recovery after the treatment **(H2)**.

Brain MRI abnormalities are common (Figure 2). Lesions involved the subcortical white matter, basal ganglia, hypothalamus, brainstem, cerebellum, meninges, ventricle, and skull. In 32 Mayo Clinic patients, 18 of 32 (56%) had T2 hyperintensities and 21 of 32 (66%) had gadolinium enhancement (3). The characteristic pattern was brain linear perivascular radial gadolinium enhancement in the white matter perpendicular to the ventricle (Figures 2A–D), which was observed in 17 patients (53%). They also found a radial enhancement pattern in two patients with cerebellum abnormalities. Other enhancement patterns were observed in leptomeningeal (*n* = 7, 22%), sinuous demyelination (*n* = 6, 19%), and ependymal (*n* = 3, 9%) regions. Iorio et al. found hyperintense lesions on T2-weighted images consistent with inflammation present in 10 of 22 patients (45%), of which nine (41%) showed gadolinium enhancement. However, no cases with a characteristic pattern with radial enhancement were described in their study (7). In Chinese patients (5), 17 of 19 showed brain abnormalities (89.5%). Radial enhancing patterns were found in eight (42.1%,) and cortical abnormalities were found in four patients (21.1%). Positron emission computed tomography results from one patient showed extensive hypermetabolism in the cortex (5) and another patient showed hypometabolism in the basal ganglia (9). Other abnormalities occurred in the hypothalamus (15.8%), midbrain (36.8%), pons (68.4%), medulla (36.8%), cerebellum (36.8%), meninges (21.1%), skull (5.3%), and hydrocephalus (5.3%). The brain enhancement disappeared soon after treatment (4). Pathology showing meningitis and inflammation around small blood vessels indicated that the enhancement was caused by gadolinium leaking from the damaged blood-brain barrier (5). Following treatment, the blood-brain barrier was repaired rapidly and the enhancement disappeared.

Myelitis is commonly seen in GFAP astrocytopathy (2–13). Among 71 patients with meningoencephalomyelitis from the Mayo Clinic, meningoencephalomyelitis phenotypes with myelitis were noted in 29 cases whereas myelitis alone was reported in two cases (combined: 43.6%, 31/71) (13). In early reports, of eight patients with MRI spine images available, six had longitudinally extensive myelitic abnormalities, one had a short myelitic lesion, and one had normal imaging. Linear-appearing central canal enhancement was noted in 21% of spinal cord magnetic resonance images (2). Abnormalities of the spinal cord were detected in four patients (4/22, 18.2%). In the study by Iorio (7), and three had a lesion that extended longitudinally for more than three contiguous vertebral segments. Recently, a study led by Sechi et al. (18) found that spinal cord lesions in GFAP-IgG myelitis were commonly longitudinally extensive (≥80%) and centrally located. Compare to AQP4-IgG lesions, they were more subtle lesions with poorly defined margins and less swelling. In GFAP-IgG myelitis, spinal cord central canal, punctate or leptomeningeal enhancement was typical. Our study revealed more common spinal cord lesions. In Chinese cases, cervical and thoracic spinal cord MRIs were performed for 16 patients (84.2%, 16/19). Thirteen of these (81.25%) exhibited abnormal results, of which 11 patients had longitudinally extensive spinal cord lesions (LESCLs) (5). All cases showed central gray matter involvement in the spinal cord (Figure 2G1). LESCLs also were reported in some single cases (8).

## Diagnosis and Overlapping Autoimmune Syndromes

Currently, there are no uniform diagnostic criteria or consensus for GFAP astrocytopathy. The following questions require answers: (1) should the diagnosis of GFAP astrocytopathy be based on the presence of GFAP antibody in the CSF or on the criteria of meningoencephalomyelitis? We should not ignore that there have been some GFAP seropositivity in disease controls (e.g., astrocytoma) and perhaps it could occur occasionally as a secondary phenomenon. Should we term it collectively as “GFAP spectrum disorders?” It seems that the “classical phenotype” is meningoencephalitis or myelomeningoencephalitis, especially with positive CSF GFAP-IgG. However, the clinical scpectrum might be broader. (2) does the diagnosis of GFAP antibody negative astrocytopathy exist in patients manifesting as typical meningoencephalomyelitis, which was previously known as non-vascular autoimmune inflammatory meningoencephalitis?; (3) how should we classify other neurologic syndromes with GFAP antibody?; and (4) does GFAP astrocytopathy usually coexist with other autoantibodies that bind to astrocytes, neurons, and oligodendrocytes? For example, coexisting neural autoantibodies were detected in five patients in the study by Iorio et al. (7). Lennon found that 41 of 102 patients (40%) had coexisting antibodies in the CSF. N-methyl-D-aspartate receptor-IgG was the most common coexisting antibody. The other coexisting antibodies included aquaporin 4-IgG, anti-neuronal nuclear antibody-1, Purkinje-cell cytoplasmic IgG, leucine-rich glioma-inactivated protein 1-IgG, contactin-associated protein 2-IgG, and glutamic acid decarboxylase-65 isoform-IgG (3). In our study, 10 patients had GFAP antibody coexisting with other specific autoantibodies (5). Therefore, overlapping antibodies are common in GFAP astrocytopathy and make the diagnosis more difficult, especially at the initial attack. Care is needed when expanding the clinical spectrum based on serum positivity given it may be overlap with other autoimmune diseases.

## Treatment and Prognosis

The treatment of GFAP astrocytopathy in the acute stage includes high-dose corticosteroids, intravenous immunoglobulin (IVIG), and plasma exchange. Long-term treatment includes oral steroids and immunosuppressants. About 70% of patients respond well to steroid therapy although some patients are prone to relapse. As a result, a standard treatment regimen has not been developed yet. According to our experience, some patients had a poor response to treatment or even died, and some patients were left with different degrees of functional disability (4).

Pathological biopsy showed that CD138^+^ plasma cells were present in the brain lesions of the patients (5). This suggested that autoantibodies were synthesized in the brain, explaining why antibodies in the CSF are higher than in the peripheral blood. Furthermore, the continuous secretion of antibodies in the brain might affect the therapeutic effect. From our retrospective experience, pure steroid or IVIG therapy has a poor effect on patients with extensive brain or spinal cord lesions as well as high concentrations of GFAP antibodies in the CSF. Plasma exchange or immunosuppressive therapy may be more beneficial in the early stages of disease. This is also in accordance with the study in GME in dogs (26).

## Future Perspectives and Conclusions

GFAP astrocytopathy is an autoimmune disease of the nervous system that requires further study regarding its etiology, pathology, mechanism, diagnosis, and treatment. In the report from the Mayo Clinic, most patients were Caucasian, while all subjects in our study were of Han nationality. Therefore, we should compare our patients with Caucasians regarding the prevalence, clinical manifestations, and prognosis.

To date, five of our patients have undergone pathological examination. However, stereotactic biopsy has some limitations. Invasive biopsy, or even autopsy, might provide more clear data regarding the immune mechanism involved in GFAP astrocytopathy. Currently, researchers consider GFAP antibody does not induce disease, but the main mechanism involved is still unknown. *In vitro* cell culture and animal experiments will enhance our understanding.

The clinical diagnosis of GFAP astrocytopathy needs to be resolved, especially in Overlapping Autoimmune Syndromes. Treatment in the acute stage includes glucocorticoids, IVIG, and plasma exchange. In addition to oral steroids the long-term use of immunosuppressive agents is appropriate for some patients.

## Author Contributions

FS and YL designed the concept, wrote the manuscript, and finalized it. WQ edited and read the final manuscript.

### Conflict of Interest Statement

The authors declare that the research was conducted in the absence of any commercial or financial relationships that could be construed as a potential conflict of interest.

## Abbreviations

GFAP: autoimmune glial fibrillary acidic protein
IF: indirect immunofluorescence
CBA: cell-based assay
NMOSD: neuromyelitis optica spectrum disorders
AQP: aquaporin
CSF: cerebrospinal fluid
NAIM: non-vasculitic autoimmune inflammatory meningoencephalitis
MRI: magnetic resonance imaging
NMDA: methyl-D-aspartic acid
GME: granulomatous meningoencephalomyelitis
NME: necrotizing meningoencephalitis
NLE: necrotizing leukoencephalitis
LESCLs: longitudinally extensive spinal cord lesions
CNS: central nervous system
IVIG: immunoglobulin
